# Supplementary material for: Energetic shifts reflect survival likelihood in Anopheles gambiae
Source: Commun Biol. 2025 Nov 12;8:1558. doi: 10.1038/s42003-025-09126-0 (PMC12612041; doi:10.1038/s42003-025-09126-0)
Supplement: Supplementary file 2 — Description of Additional Supplementary Files [file 42003_2025_9126_MOESM2_ESM.pdf]

Description of Additional Supplementary Files

File: Supplementary Data 1

Description: Data used in the study.

File: Supplementary Data 2

Description: R Script for the analyses run and figures generated in the study.
