# Supplementary material for: Energetic shifts reflect survival likelihood in Anopheles gambiae
Source: Commun Biol. 2025 Nov 12;8:1558. doi: 10.1038/s42003-025-09126-0 (PMC12612041; doi:10.1038/s42003-025-09126-0)
Supplement: Supplementary file 5 — Reporting Summary [file 42003_2025_9126_MOESM5_ESM.pdf]

nature portfolio

Corresponding author(s): Luis Silva

Last updated by author(s): Oct 16, 2025

Reporting Summary

Nature Portfolio wishes to improve the reproducibility of the work that we publish. This form provides structure for consistency and transparency in reporting. For further information on Nature Portfolio policies, see our Editorial Policies and the Editorial Policy Checklist.

Statistics

For all statistical analyses, confirm that the following items are present in the figure legend, table legend, main text, or Methods section.

n/a	Confirmed	
☐	☒	The exact sample size (*n*) for each experimental group/condition, given as a discrete number and unit of measurement
☐	☒	A statement on whether measurements were taken from distinct samples or whether the same sample was measured repeatedly
☐	☒	The statistical test(s) used AND whether they are one- or two-sided<br>*Only common tests should be described solely by name; describe more complex techniques in the Methods section.*
☐	☒	A description of all covariates tested
☐	☒	A description of any assumptions or corrections, such as tests of normality and adjustment for multiple comparisons
☐	☒	A full description of the statistical parameters including central tendency (e.g. means) or other basic estimates (e.g. regression coefficient) AND variation (e.g. standard deviation) or associated estimates of uncertainty (e.g. confidence intervals)
☐	☒	For null hypothesis testing, the test statistic (e.g. *F*, *t*, *r*) with confidence intervals, effect sizes, degrees of freedom and *P* value noted<br>*Give P values as exact values whenever suitable.*
☒	☐	For Bayesian analysis, information on the choice of priors and Markov chain Monte Carlo settings
☒	☐	For hierarchical and complex designs, identification of the appropriate level for tests and full reporting of outcomes
☒	☐	Estimates of effect sizes (e.g. Cohen's *d*, Pearson's *r*), indicating how they were calculated

Our web collection on statistics for biologists contains articles on many of the points above.

Software and code

Policy information about availability of computer code

Data collection	NA
Data analysis	Only R was used for statistics.

For manuscripts utilizing custom algorithms or software that are central to the research but not yet described in published literature, software must be made available to editors and reviewers. We strongly encourage code deposition in a community repository (e.g. GitHub). See the Nature Portfolio guidelines for submitting code & software for further information.

Data

Policy information about availability of data

All manuscripts must include a data availability statement. This statement should provide the following information, where applicable:

- Accession codes, unique identifiers, or web links for publicly available datasets
- A description of any restrictions on data availability
- For clinical datasets or third party data, please ensure that the statement adheres to our policy

All data and R scripts will be provided as supplementary information of this manuscript.

Research involving human participants, their data, or biological material

Policy information about studies with human participants or human data. See also policy information about sex, gender (identity/presentation), and sexual orientation and race, ethnicity and racism.

Reporting on sex and gender	Not applicable
Reporting on race, ethnicity, or other socially relevant groupings	Not applicable
Population characteristics	Not applicable
Recruitment	Not applicable
Ethics oversight	Not applicable

Note that full information on the approval of the study protocol must also be provided in the manuscript.

Field-specific reporting

Please select the one below that is the best fit for your research. If you are not sure, read the appropriate sections before making your selection.

☐ Life sciences ☐ Behavioural & social sciences ☒ Ecological, evolutionary & environmental sciences

For a reference copy of the document with all sections, see nature.com/documents/nr-reporting-summary-flat.pdf

Ecological, evolutionary & environmental sciences study design

All studies must disclose on these points even when the disclosure is negative.

Study description	In this study we quantified resource and energy dynamics of the major macro-resources used by mosquitoes under stress. As stressors we had a) infection, or not; b) blood meal, or not. Our findings show the lifetime dynamics of Anopheles gambiae under the different stresses and demonstrates that mosquitoes set to survive, have an energetic shift that mosquitoes set to die do not.
Research sample	Whole body mosquitoes
Sampling strategy	Live individuals were collected at five-day intervals starting on day 7 (before the bloodmeal) and continuing through day 32. Dead individuals were collected every 12 hours as they naturally died. The collected mosquitoes were frozen at – 80ºC until we assayed spores, proteins, carbohydrates and lipids.
Data collection	LMS and TGZ collected the data
Timing and spatial scale	Samples were collected between March 2022 and July 2022. All measurements were done between July 2022 and November 2022.
Data exclusions	No data was excluded from any analyses.
Reproducibility	Use the same exact host-pathogen genotype, meaning Anopheles gambiae strain Kisumu as host, and Vavraia culicis, subs. floridensis as a parasite. Use the same reagents and stick to the same exact collection time-points.
Randomization	All mosquitoes were kept in a controlled temperature and humidity room and all treatments were equally and randomly dispersed throughout the room until sample collection. Samples were processed without knowing which treatment they belonged for all the measurements.
Blinding	Samples were processed without knowing which treatment they belonged for all the measurements. A numerical key was used to know which treatment the sample belonged to but the authors were only informed of the treatment upon statistical analysis.

Did the study involve field work? ☐ Yes ☒ No

Reporting for specific materials, systems and methods

We require information from authors about some types of materials, experimental systems and methods used in many studies. Here, indicate whether each material, system or method listed is relevant to your study. If you are not sure if a list item applies to your research, read the appropriate section before selecting a response.

Materials & experimental systems

n/a	Involved in the study
☒	☐ Antibodies
☒	☐ Eukaryotic cell lines
☒	☐ Palaeontology and archaeology
☐	☒ Animals and other organisms
☒	☐ Clinical data
☒	☐ Dual use research of concern
☒	☐ Plants

Methods

n/a	Involved in the study
☒	☐ ChIP-seq
☒	☐ Flow cytometry
☒	☐ MRI-based neuroimaging

Animals and other research organisms

Policy information about studies involving animals; ARRIVE guidelines recommended for reporting animal research, and Sex and Gender in Research

Laboratory animals	Anopheles gambiae (sensu stricto) laboratory strain Kisumu
Wild animals	We only used a laboratory population of mosquitoes.
Reporting on sex	Sex was considered in this study. Given that only females are able to take a blood meal (to reproduce), then we only considered female in this study.
Field-collected samples	We only used a laboratory population of mosquitoes.
Ethics oversight	Both the host (Anopheles gambiae) and parasite (Vavraia culicis) do not require ethical concerns.

Note that full information on the approval of the study protocol must also be provided in the manuscript.

Plants

Seed stocks	Not applicable to this study.
Novel plant genotypes	Not applicable to this study.
Authentication	Not applicable to this study.
